# Preventative effect of TSPO ligands on mixed antibody-mediated rejection through a Mitochondria-mediated metabolic disorder

**DOI:** 10.1186/s12967-023-04134-2

**Published:** 2023-05-02

**Authors:** Yannan Zhang, Jiannan He, Zhe Yang, Haofeng Zheng, Haoxiang Deng, Zihuan Luo, Qipeng Sun, Qiquan Sun

**Affiliations:** 1 Medical Research Institute, Guangdong Provincial People’s Hospital (Guangdong Academy of Medical Sciences), Southern Medical University, Guangzhou, China; 2 Division of kidney Transplantation, Guangdong Provincial People’s Hospital (Guangdong Academy of Medical Sciences), Southern Medical University, 106 2nd road Zhongshan, Yuexiu District, Guangzhou, 510080 China; 3 Department of Urology, The Sixth Affiliated Hospital, Sun Yat-sen University, Guangzhou, China; 4 Department of Urology, The First Affiliated Hospital of Shandong First Medical University, Jinan, China

**Keywords:** TSPO ligand, AMR, Heart transplantation, Cell metabolism

## Abstract

**Background:**

Immune-mediated rejection was the major cause of graft dysfunction. Although the advances in immunosuppressive agents have markedly reduced the incidence of T-cell-mediated rejection after transplantation. However, the incidence of antibody-mediated rejection (AMR) remains high. Donor-specific antibodies (DSAs) were considered the major mediators of allograft loss. Previously, we showed that treatment with 18-kDa translocator protein (TSPO) ligands inhibited the differentiation and effector functions of T cells and reduced the rejection observed after allogeneic skin transplantation in mice. This study we further investigate the effect of TSPO ligands on B cells and DSAs production in the recipients of mixed-AMR model.

**Methods:**

In vitro, we explored the effect of treatment with TSPO ligands on the activation, proliferation, and antibody production of B cells. Further, we established a heart-transplantation mixed-AMR model in rats. This model was treated with the TSPO ligands, FGIN1-27 or Ro5-4864, to investigate the role of ligands in preventing transplant rejection and DSAs production in vivo. As TSPO was the mitochondrial membrane transporters, we then investigated the TSPO ligands effect on mitochondrial-related metabolic ability of B cells as well as expression of downstream proteins.

**Results:**

In vitro studies, treatment with TSPO ligands inhibited the differentiation of B cells into CD138^+^CD27^+^ plasma cells; reduced antibodies, IgG and IgM, secretion of B cells; and suppressed the B cell activation and proliferation. In the mixed-AMR rat model, treatment with FGIN1-27 or Ro5-4864 attenuated DSA-mediated cardiac-allograft injury, prolonged graft survival, and reduced the numbers of B cells, including IgG^+^ secreting B cells, T cells and macrophages infiltrating in grafts. For the further mechanism exploration, treatment with TSPO ligands inhibited the metabolic ability of B cells by downregulating expression of pyruvate dehydrogenase kinase 1 and proteins in complexes I, II, and IV of the electron transport chain.

**Conclusions:**

We clarified the mechanism of action of TSPO ligands on B-cell functions and provided new ideas and drug targets for the clinical treatment of postoperative AMR.

**Supplementary Information:**

The online version contains supplementary material available at 10.1186/s12967-023-04134-2.

## Introduction

Immune rejection, including cell-mediated rejection (CMR) and antibody-mediated rejection (AMR), due to incompatibility with the human leukocyte antigen system, is a major obstacle to successful organ transplantation [1]. Immunosuppressants reduce CMR significantly, but have weak efficacy against AMR [2, 3]. ​AMR usually occurs when recipients are pre-sensitized before surgery or develop de novo donor-specific antibodies (DSAs) after surgery. High levels of donor-specific IgG in serum and C4d deposition in the interstitial vasculature are considered the best markers of AMR [4].

The therapeutic approaches to AMR involve removing antibodies and/or eliminating B cells, including intravenous immunoglobulin, plasma exchange, and rituximab [1]. However,​ these methods were only modestly effective. Intravenous immunoglobulin and plasma exchange have had limited success due to antibody rebounding. Rituximab targets CD20 to deplete B cells. But plasma cells, which produce DSAs, do not express CD20, limiting the effectiveness of this approach [5–7]. The challenge of suppressing B cell functions and managing DSAs in organ transplantation has been the urgent priorities to be addressed. The development of new strategies is needed.

18-kDa translocator protein (TSPO) is a conserved protein located in the outer mitochondrial membrane [8] [9] expressed widely in different types of immune cells (e.g., monocytes, T cells, B cells, other subsets) to link to the immune response. The TSPO ligands Ro5-4864 and PK11195 inhibit the production of interleukin (IL)-1β, IL-6, and tumor necrosis factor-α in macrophages [10] and relieve nerve inflammation when moderating the severity of multiple sclerosis [11]. Previously, we showed that treatment with TSPO ligands, FGIN1-27 or Ro5-4864, inhibited the differentiation and cytokine production of human T-helper type-1 cells, and suppressed allograft rejection in a murine-skin transplant model by inhibiting infiltration by inflammatory cells and interferon-γ production [12]. TSPO is also expressed in B cells, but relevant research has not been conducted.

Metabolism plays a crucial role in immune cells. Previous studies have confirmed that when immune cells are activated, their energy provided from metabolism must be raised up to satisfied cell vitality and play the effector function [13, 14]. Mitochondria are key organelles for energy production, and TSPO is mainly located in the mitochondrial outer membrane and is a key regulator of mitochondrial homeostasis [15]. Previous studies have shown that TSPO deficiency can inhibit the mitochondrial function of microglia cells and significantly reduce the levels of mitochondrial oxidative phosphorylation (OXPHOS) and glycolysis, thus inhibiting microglia activation [16].

In this study, we confirmed that TSPO ligands, FGIN1-27 and Ro5-4864, inhibited the activation and proliferation of B cells. Also, TSPO ligands constrained the B cells differentiated into plasma cells and the antibodies, IgG and IgM, production *in vitro. In vivo*, we established a mixed antibody-mediated rejection model in rat hearts. TSPO ligands prolonged the duration of survival of cardiac allografts, reduced infiltration by inflammatory cells and relieved the circulating DSAs and deposition of the complement component C4d in grafts. TSPO ligands inhibited the mitochondrial-related metabolic ability of B cells by downregulating expression of pyruvate dehydrogenase kinase (PDK)1 and proteins in the electron transport chain (ETC) complexes I, II, and IV. This study may provide new ideas and drug targets for the clinical treatment of postoperative AMR.

## Methods

### Animal and human materials

Animal experiments were undertaken in the Guangdong Province Animal Center (Guangdong, China). The study protocol was approved (KY-Q-2022-157-01) by the Animal Care and Use Committee of Guangdong Province People’s Hospital (Guangdong, China).

Male Brown Norway (BN) rats and Lewis rats (200–250 g) were purchased from Beijing Vital River Laboratory Animal Technology (Beijing, China). Peripheral-blood mononuclear cells (PBMCs) were obtained from Oricell (Shanghai, China).

### B-cell cultures in vitro

CD19^+^ B cells were purified by anti-CD19-positive magnetic beads (Miltenyi Biotec, Bergisch Gladbach, Germany) from human PBMCs and the purity was consistently > 95% (as determined by flow cytometry). B cells were cultured with CpG plus anti-IgM (MilliporeSigma) in the presence or absence of TSPO ligands, FGIN1-27 (Tocris, Baldwin, MO, USA) or Ro5-4864 (MilliporeSigma, Burlington, MA, USA) in an atmosphere of 5% CO_2_ at 37 °C.

For detecting the secretion of immunoglobulin, CD19^+^ B cells were cultured for 9 days. The supernatants were harvested, and the concentrations of IgG and IgM were measured by ELISA.

For detecting the activation and proliferation, CD19^+^ B cells were cultured for 2 days. The activated molecule CD25 and CD69 were measured by FACs, and the proliferative capacity was evaluated by BrdU incorporation assay.

### Enzyme-linked immunosorbent assay (ELISA)

Supernatants were harvested at day 9. The concentrations of IgG and IgM were measured using ELISA kits (BD Bioscience, San Jose, CA, USA) according to manufacturer instructions.

### Brdu incorporation assay

Purified B cells were cultured for 2 days and the BrdU was added at the final 1 h of incubation. The cells were stained with the FITC-conjugated anti-BrdU according to the BrdU incorporation assay (FITC BrdU Flow kit, BD Bioscience).

### Transplant surgery and treatment

The BN rat was the donor, and the Lewis rat was the recipient. Inhalation anesthesia was undertaken with isoflurane (~ 2%). For skin transplantation, the tail skin graft (1.5–2.0 × 2 cm) from the donor was transplanted onto the dorsal area of the recipient. For the mixed AMR model, recipient rats received the donor cardiac allograft 14 days after skin graft pre-sensitization. Cyclosporin A (CSA) was injected subcutaneously at a dose of 20 mg/kg/day, from day 0 after skin transplantation to allo-heart loss or obtained for detection.

The detailed processes of cardiac transplantation have been described previously [17, 18]. Briefly, a graft from a donor heart was harvested and transplanted into the abdominal cavity of a recipient rat by anastomosing the aorta and pulmonary artery of the graft end-to-side to the aorta and vena cava, respectively, of the recipient. Survival of the cardiac graft was defined as production of a heartbeat. Cardiac arrest denoted rejection of the cardiac graft. Rats were euthanized at the day 4 after heart transplantation of organ extraction or at the endpoint of observation with an overdose of anesthetics and neck dislocation.

The simply heard transplantation without pre-sensitized with allo-skin was mainly recognized as acute cellular rejection. Compared with the non-sensitized, this pre-sensitized model exhibited a typical AMR presentation, highly level of circulating-DSAs and C4d deposition in the graft (Additional file 1: Fig S1A–B).

### Animal experimental protocol

For experiments based on the skin transplantation and cardiac AMR transplantation, recipients were assigned randomly to three groups: [1] FGIN1-27 (1 mg/kg/day); [2] Ro5-4864 (1 mg/kg/day); [3] control (equal volume of physiologic (0.9%) saline daily). The FGIN1-27, Ro5-4864, and control groups were treated every day from day 0 till the animal is euthanized or dies of natural. The drugs were administration orally to the animals.

### Assay to determine the circulating level of donor-specific antibodies (DSAs)

Circulating levels of DSAs were detected by flow cytometry. Sera were collected at the indicated time (in skin transplantation model: every four days from 0 to 36 days after skin transplantation; in heart transplantation model: 4 days after cardiac transplantation) from the orbit of the recipient rat. Sera were incubated with donor splenocytes for 30 min at 37 °C. After washing twice with phosphate-buffered saline, splenocytes linked with DSAs were incubated with anti-rat FITC-IgM and Percpcy5.5-IgG for 1 h at 4 °C. Cells were analyzed by flow cytometry, and the results are shown as the mean fluorescence intensity.

### Histology and IHC analyses

Cardiac grafts were obtained 4 days after cardiac transplantation. Tissues were embedded in paraffin after fixing with 4% paraformaldehyde overnight. Then, embedded tissues were cut into sections of thickness 3 μm and stained with hematoxylin and eosin (H and E) and the corresponding primary antibodies. Antibodies against cluster of differentiation (CD)3, CD4, CD8, CD68, and C4d in rats used for IHC staining were purchased from Abcam (Cambridge, UK). A Horseradish Peroxidase Kit (Beyotime Institute of Biotechnology, Shanghai, China) and 3,3′-diaminobenzidine (Dako, Carpinteria, CA, USA) were employed for chromogen visualization. The average optical density (AOD) was measured by image pro plus for the quantitative analysis of the positive stain.

### Terminal deoxynucleotidyl transferase (TdT)-Mediated dUTP Nick-End labeling (TUNEL) staining

​Paraffin sections were prepared by de-paraffinization and rehydration, and then subjected to TUNEL staining using a one-step TUNEL Apoptosis assay kit (Beyotime Institute of Biotechnology). DAPI staining was used for visual observation.

### Flow cytometry

For the detection of cell activation in vitro, purified CD19^+^ B cells were cultured for 2 days and incubated with APC-CD25 and PE-CD69 (BD Bioscience) for 30 min at 4 °C in dark.

For the detection of lymphocytes in the model, peripheral blood, fresh cardiac grafts and recipient spleens were obtained on 4 days after cardiac transplantation, before the allo-graft failure. Fresh recipient cardiac grafts were digested in phosphate-buffered saline supplemented with 1% heat-inactivated fetal bovine serum with collagenase 1 and DNase for 60 min at 37 °C, before pressing through a 200 mesh nylon screen. The collected cells were isolated by gradient density centrifugation using ficoll-paque premium (Cytiva, Washington, USA). Collected lymphocytes were stained with fluorescently labeled antibodies. Pacificblue-CD45, PE-CD45R, fluorescein isothiocyanate (FITC)-CD3, APCcy7-CD4, Percp-CD8, PEcy7-CD11b, APC-CD161, FITC-immunoglobulin (Ig)M, and Percpcy5.5-IgG antibodies used for flow cytometry were from BD Biosciences.

Detection was determined by a flow cytometer (CytoFLEX; Beckman Coulter, Fullerton, CA, USA) and data were analyzed by FlowJo (Tree Star, Ashland, OR, USA).

### Seahorse XF assay

Extracellular acidification rate (ECAR) and oxygen consumption (OCR) were determined using a Seahorse XF-96 Extracellular Flux Analyzer (Seahorse Bioscience, Agilent Technologies, CA, US). An XF96 cell-culture microplate was coated with Cell-Tak^™^ before inoculation with B cells, and B cells were seeded at 4 × 10^5^ cells/well in plates. ​The assay medium was unbuffered RPMI-1640 (with 10 mM glucose, 1 mM pyruvate, and 2 mM L-glutamine at pH 7.4). Baseline ECAR and OCR and their response to the indicated compounds (2.5 mM oligomycin, 1.5 mM FCCP, 500 nM rotenone, and 500 nM antimycin A) were determined (all from Seahorse Bioscience). Pathway analysis was performed by the clusterProfiler package.

### Western blotting

Purified human CD19^+^ B cells were stimulated with anti-IgM plus CpG with or without FGIN1-27 or Ro5-4864 for 48 h and lysed in lysis buffer (Beyotime Institute of Biotechnology). The total proteins were quantified by BCA protein quantitation kit (Thermos Fisher Scientific, Waltham, MA, USA). Samples were separated by 10% sodium dodecyl sulfate–polyacrylamide gel electrophoresis and blotted onto polyvinylidene fluoride (PVDF) membranes (Millipore, Waltham, MA, USA). PVDF membranes were blocked with 5% (w/v) non-fat milk (TBS) and incubated with the primary antibodies against pyruvate dehydrogenase kinase 1 (PDK-1) (MW:47KD; antibody dilute: 1:1000), succinate dehydrogenase subunit alpha (SDHA) (MW:70KD; antibody dilute: 1:1000), NADH-ubiquinone oxidoreductase chain 1 (ND1) (MW:36KD; antibody dilute: 1:1000), cytochrome C oxidase subunit 1 (COX1) (MW:68KD; antibody dilute: 1:1000), cytochrome B (CYTB) (MW:26KD; antibody dilute: 1:1000), and β-actin (Abcam) (MW:42KD; antibody dilute: 1:1000) diluted by antibody dilution (5% (w/v) BSA + TBS). After incubation with the relevant secondary antibodies (antibody dilute: 1:2000), PVDF membranes were visualized using the ECL Western Blotting Analysis System (GE AI600; GE Healthcare, Fairfield, CT, USA).

### Real-time reverse transcription-quantitative polymerase chain reaction (RT-qPCR)

Transcript levels were determined using real-time RT-qPCR. RNA from tissues or cells was extracted with Rnase Micro Kit (Qiagen, Hilden, Germany) according to manufacturer protocols. For reverse transcription, PrimeScript RT Master Mix (Roche, Basel, Switzerland) was used. For qPCR, SYBR Green I Master Mix (Roche) was employed, and the cytoskeletal gene *β-actin* was used for the normal control. The primer sequences we used are shown in Table 1.

**Table 1:** Primers used for qPCR

Gene	Forward (5′–3′)	Reverse (5′–3′)
*PDK1*	CTGTGATACGGATCAGAAACCG	TCCACCAAACAATAAAGAGTGCT
*PDHA1*	ATGGAATGGGAACGTCTGTTG	CCTCTCGGACGCACAGGATA
*PDHA2*	GGGCGGAGGGGCTTAAATAC	GTGACAGAAACCGCGAATGAA
*CS*	TGCTTCCTCCACGAATTTGAAA	CCACCATACATCATGTCCACAG
*IDH1 (NADP+)*	TGTGGTAGAGATGCAAGGAGA	TTGGTGACTTGGTCGTTGGTG
*OGDH*	GGCTTCCCAGACTGTTAAGAC	GCAGAATAGCACCGAATCTGTTG
*ND1 (NDUFS1)*	TGGAAGACAAGAACATTGGGC	GCAAACCTGATGCAGCGAG
*SDHA*	TGGCATTTCTACGACACCGTG	GCCTGCTCCGTCATGTAGTG
*CYTB*	GCTGTTATGTACCCAAGCAAAGA	TCCCCACTCAATTCCATCACT
*COX1*	AACCCAATACCAAACGC	CTTCAGGGTGACCGAAA
*β-actin*	CATGTACGTTGCTATCCAGGC	CTCCTTAATGTCACGCACGAT

### Statistical analyses

For comparison of the data from two groups, we used the Student’s *t*-test to analyze significant differences. Graft survival was evaluated by the log-rank test. Data are the mean ± standard deviation. Statistical analyses were carried out by Prism (GraphPad, La Jolla, CA, USA). *P* < 0.05 and *P* < 0.01 were considered significant.

## Results

### TSPO ligands inhibit the differentiation and antibody secretion of B cells *in vitro*

We first verify the expression of TSPO on B cells. As it shown in Additional file 1: Fig S2, both rest and activated CD19 + B cells expressed TSPO. We further assessed the effect of TSPO ligands on B cells by carrying out experiments on B-cell cultures. To exclude the possibility that TSPO ligands inhibit B-cell function by promoting apoptosis, purified B cells were cultured with different concentrations of FGIN1-27 or Ro5-4864 for 48 h. Staining (annexin V/propidium iodide) was undertaken to evaluate cell survival. Treatment with FGIN1-27 or Ro5-4864 had little effect on B-cell survival at concentrations of 10 µM to 100 µM (Additional file 1: Fig S3). We chose a concentration of 50 µM for subsequent in vitro experiments.

**Fig. 1:**
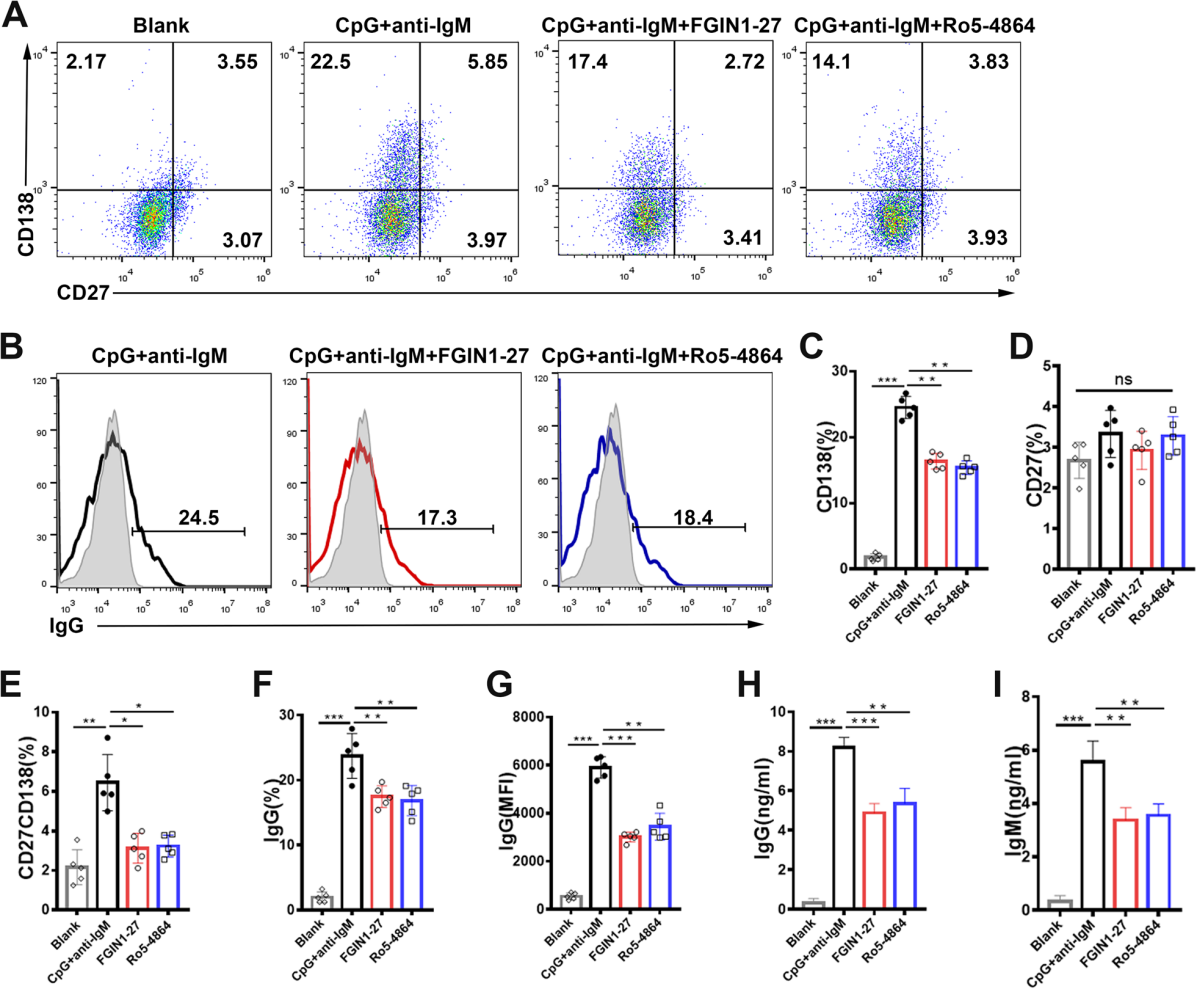
TSPO ligands suppress plasma-cell formation and antibody secretion in vitro. Purified CD19^+^ B cells were stimulated with CpG + anti-IgM, CpG + anti-IgM + FGIN1-27, or CpG + anti-IgM + Ro5-4864, for 9 days. **A**–**D** Representative flow-cytometry results and bar graphs of flow cytometry of CD138^+^ cells, CD27^+^ cells, and CD138^+^CD27^+^ cells in CD19^+^ B cells. **E**–**G** Representative flow-cytometry results and bar graphs of CD19^+^ B cells. **H**, **I** Concentrations of IgG and IgM in the culture supernatant were determined by ELISA. Data are the mean ± SD of five independent samples. n = 5; *P < 0.05; **P < 0.01; ns: no significant difference

Next, we investigated the effect of TSPO ligands on the differentiation and antibody secretion of B cells. Human CD19^+^ B cells were cultured in the presence of FGIN1-27, Ro5-4864, or equal volume solvent (dimethyl sulfoxide), and exposed to CpG plus anti-IgM for 9 days. Expression of CD27, CD138, and IgG was measured by flow cytometry. Treatment with FGIN1-27 or Ro5-4864 significantly reduced the differentiation of CD138^+^ plasma cells, CD27^+^CD138^+^ memory plasma cells, and IgG secretory B cells from naïve B cells (Fig. 1A–G). Moreover, the concentrations of IgG and IgM in the supernatant were evaluated by ELISA at day 9. Levels of IgG and IgM in FGIN1-27- and Ro5-4864-treated cells were markedly lower than those in control cells (Fig. 1H, I). These data indicated that treatment with FGIN1-27 or Ro5-4864 inhibited the differentiation and antibody secretion of B cells significantly in vitro.

### TSPO ligands reduce the proliferation and activation of B cells

**Fig. 2:**
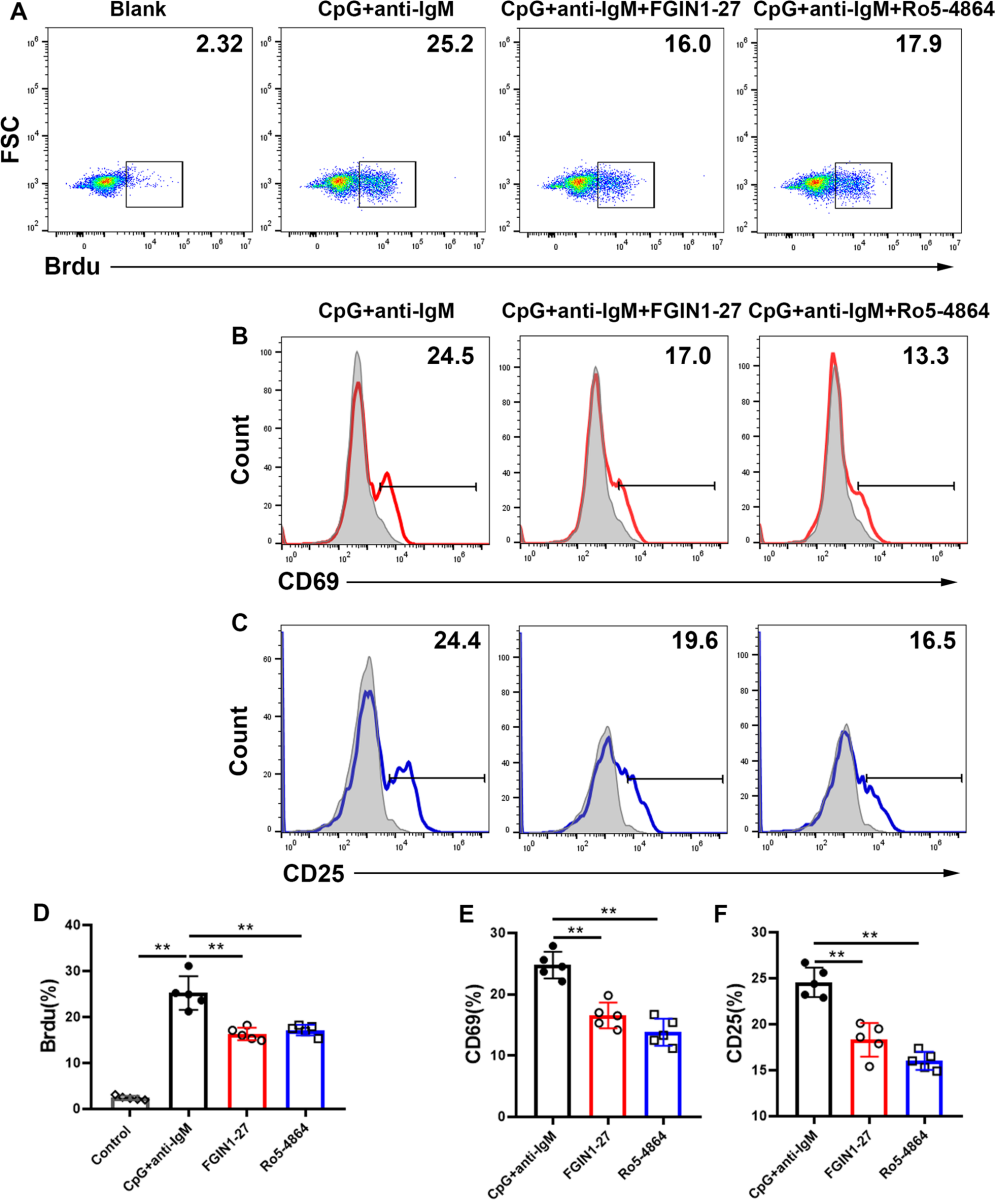
TSPO ligands reduce the proliferation and activation of B cells Purified CD19^+^ B cells were stimulated with CpG + anti-IgM, CpG + anti-IgM + FGIN1-27, or CpG + anti-IgM + Ro5-4864, for 2 days. **A**, **D** Representative bromodeoxyuridine expression and bar graphs of flow-cytometry data. **B**, **E** and (**C**, **F**) Representative expression of CD69 and CD25 and bar graphs of flow-cytometry results. Data are the mean ± SD of five independent samples. n = 5; *P < 0.05; **P < 0.01

With respect to the proliferation and activation of B cells, we used the label bromodeoxyuridine (Brdu) as well as the markers CD25 and CD69 to evaluate them. After culture for 2 days, the number of brdu-labeled cells was reduced significantly after treatment with TSPO ligands compared with the number in the control group (Fig. 2A, D). Expression of an early (CD69) and late (CD25) marker of activation was also obviously lower in the TSPO treatment group than that in the control group (Fig. 2B, C, E, F).

### TSPO ligands inhibit DSA generation in vivo

**Fig. 3:**
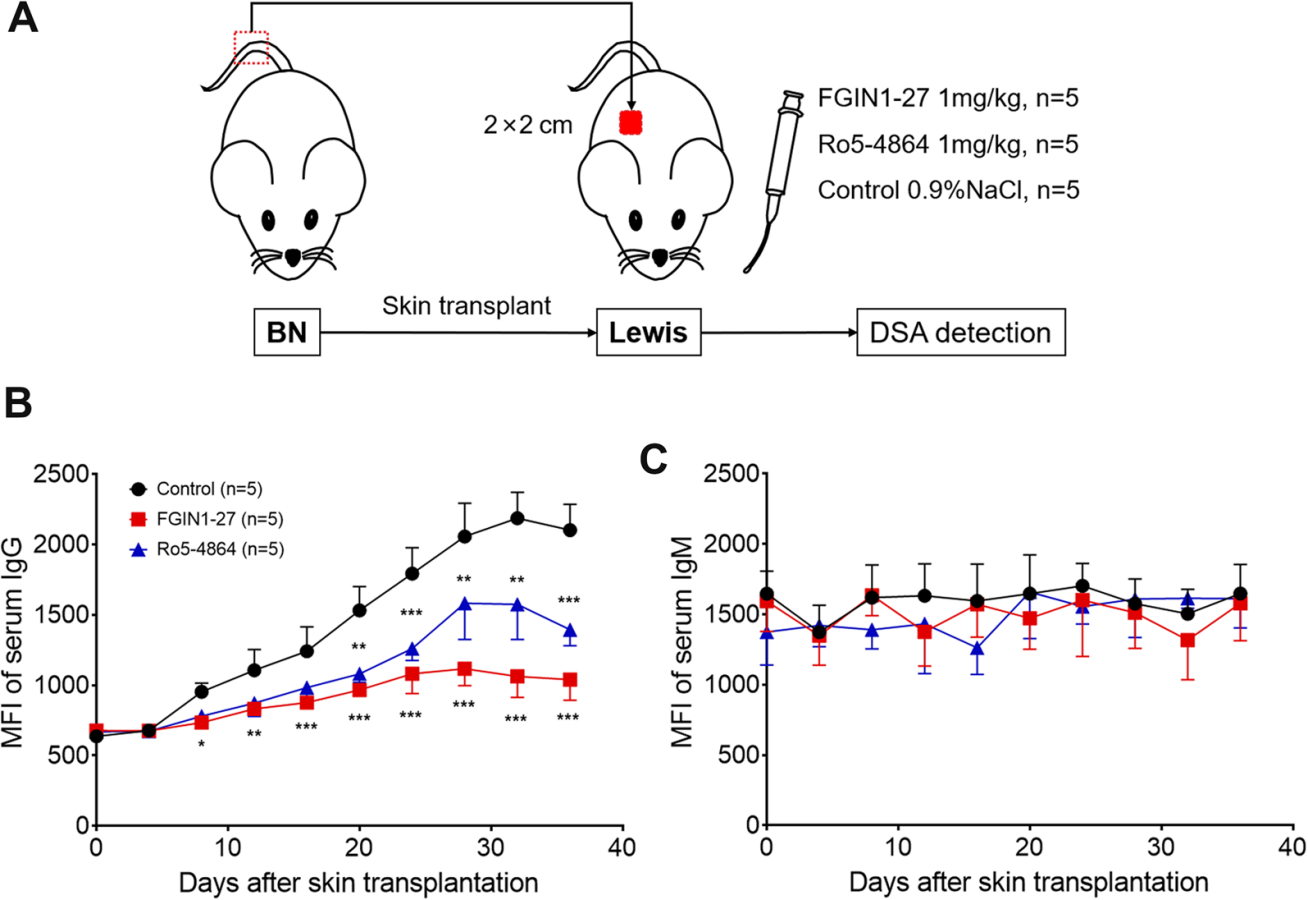
The TSPO ligands FGIN1-27 and Ro5-4864 inhibit donor-specific IgG secretion in vivo. The recipients (Lewis rats) after skin transplantation were divided into three groups, FGIN1-27, Ro5-4864, and control, who were administered FGIN1-27 (1 mg/kg), Ro5-4864 (1 mg/kg), or an equal volume of physiologic (0.9%) saline, respectively by gavage every day for 36 days. Sera from the orbit of recipient rats were collected and incubated with donor spleen cells. Levels of donor-specific IgG and IgM were measured by flow cytometry. **A** Experimental protocol of skin transplantation. **B**, **C** Circulating DSA levels were determined as the mean fluorescence intensity (MFI) ± SD (n = 5). *P < 0.05; **P < 0.01; ***P < 0.001

To investigate whether DSAs were affected by TSPO ligands, we first used a skin-graft model in rats (Fig. 3A). Lewis rats received the tail skin of donors (BN rats) and were treated with FGIN1-27, Ro5-4864, or vehicle. From day-4 to day-30 after skin transplantation, the circulating levels of IgG of recipients increased gradually and significantly. Compared with the control group, the FGIN1-27 and Ro5-4864 groups had a significant reduction in the upward trend of the IgG level (Fig. 3B). There was no upward trend in the IgM level, which indicated a low responsiveness to skin grafts, and the circulating IgM level in the FGIN1-27 and Ro5-4864 groups showed no significant difference with that in the control group (Fig. 3C). Therefore, rats treated with TSPO ligands showed lower donor-specific IgG (but not IgM) production, in the skin-transplantation model we created.

### TSPO ligands attenuate DSA-mediated cardiac-allograft injury and prolong graft survival

**Fig. 4:**
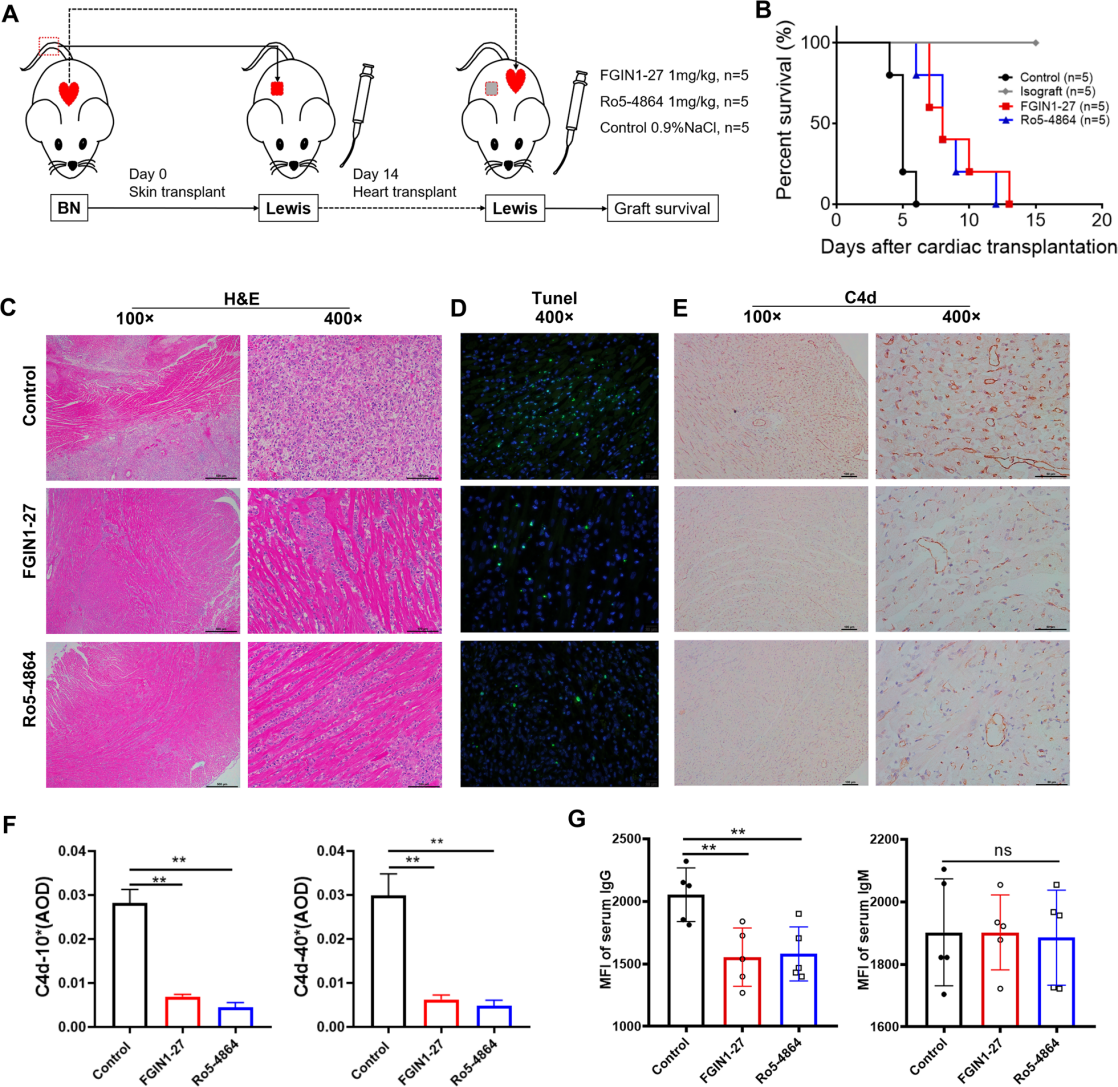
TSPO ligands attenuate DSA-mediated injury and prolong the survival of cardiac allografts. **A** Experimental protocol of creation of a model of antibody-mediated cardiac allograft rejection. **B** Survival curve of a cardiac allograft. Representative images of (**C**) hematoxylin and eosin (**H** and **E**), (**D**) TUNEL, and C4d (**E**) staining of cardiac allografts 4 days after cardiac transplantation. **F** Average Optical Density (AOD) of C4d staining. **G** Circulating levels of DSAs, IgG, and IgM were determined by flow cytometry (n = 5). **E** *P < 0.05; **P < 0.01

To further understand the effect of TSPO ligands in vivo, we established a major histocompatibility complex (MHC) mismatch model of heart transplantation in rats. Recipients (Lewis) rats were pre-sensitized with the tail skins of BN rats for 14 days before transplantation with BN hearts (Fig. 4A), and treated with vehicle, FGIN1-27, or Ro5-4864. All recipients received CSA on the day of skin transplantation to reduce the interference caused by the infiltration of T cells, as well as to retain and magnify the effect of B cells and DSAs. An isograft group with the hearts of Lewis rats transplanted into recipients (Lewis rats) was also established.

In the isograft group, all transplanted hearts survived > 15 days, which indicated that the allograft was tolerated. The mean duration of graft survival in the AMR control group was (5.0 ± 0.7) days, whereas it was (9.0 ± 2.5) days in the FGIN1-27 group and (8.6 ± 2.2) days in the Ro5-4864 group. Therefore, TSPO ligands prolonged the survival of transplanted hearts markedly in a DSA-mediated model in rats (n = 5; *P* < 0.01) (Fig. 4B).

For the further examination, we performed another set of models (n = 5). The cardiac graft and serum from circulating blood were obtained four days after heart transplantation when the allo-hearts of three groups have not been lost. In the cardiac graft of control group, H&E staining revealed severe interstitial hemorrhage, infiltration by inflammatory cells, tissue edema, and focal liquefaction and necrosis in tissue. In the groups of TSPO-ligand treatment, the number of these lesions was reduced significantly (Fig. 4C). We also undertook terminal deoxynucleotidyl transferase dUTP nick end labeling (TUNEL) staining for apoptotic cells in the graft: TSPO treatment attenuated the number of tissue lesions significantly, with fewer apoptotic cells observed compared with the control group (Fig. 4D). C4d deposited, the specific marker for acute of AMR diagnosis, was shown intense and diffuse in capillaries in control group. However, FGIN1-27 and Ro5-4864 treatment significantly attenuated the C4d deposition in the graft capillaries (Fig. 4E–F). Flow cytometry showed that the serum donor-specific IgG levels in the FGIN1-27 and Ro5-4864 groups were approximately 25% lower than those in the control group (P < 0.01), but significant differences in IgM levels were not observed (Fig. 4G).

### TSPO ligands reduce the number of lymphocytes and B cells in the cardiac allograft and host spleen

**Fig. 5:**
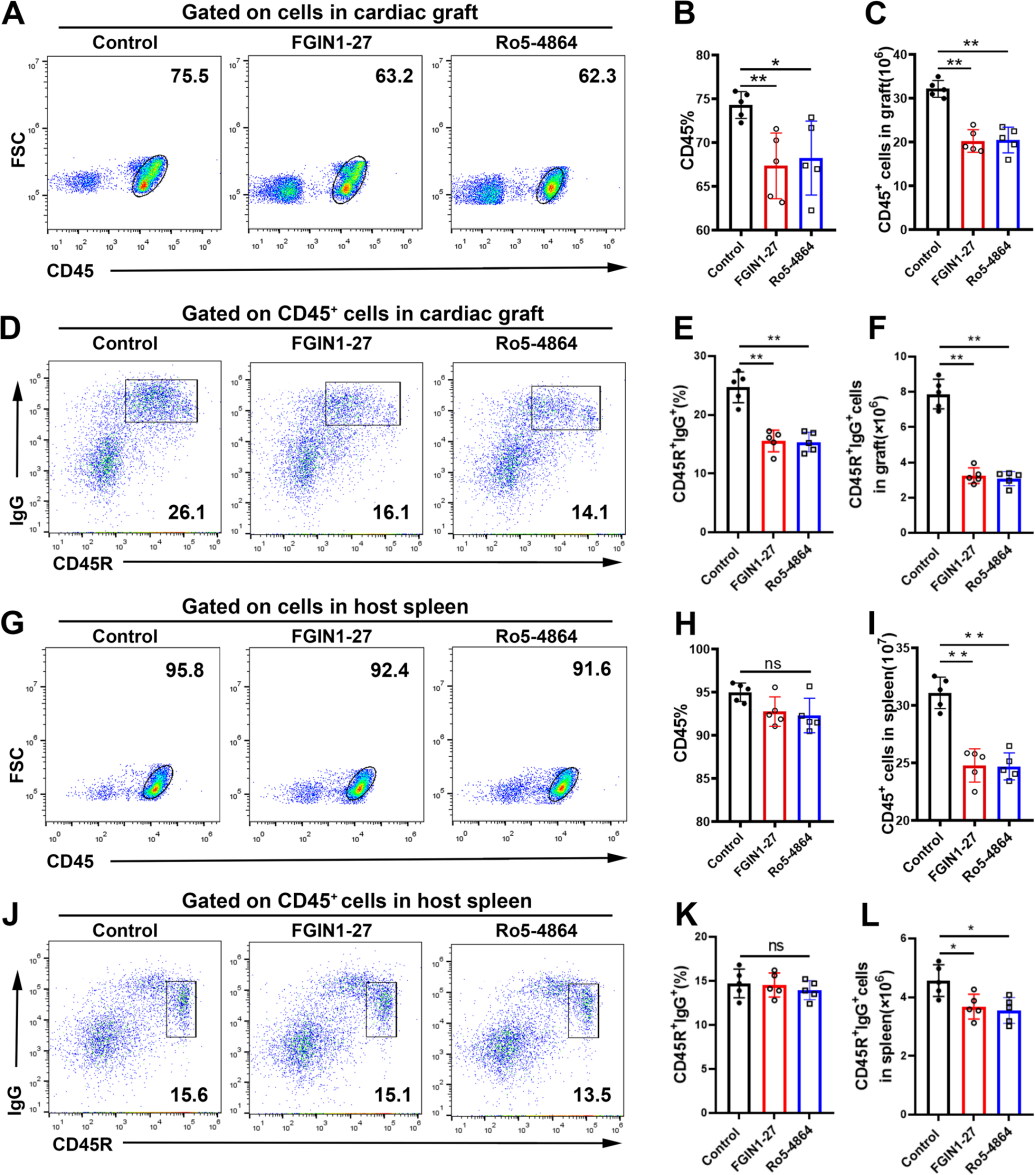
TSPO ligands reduce the infiltration of lymphocytes and B cells in a cardiac allograft and host spleen. Infiltration by lymphocytes in allografts and lymphocytes from the peripheral circulation into the spleen were determined by flow cytometry 4 after heart transplantation in the model. CD45^+^ cells were determined as the lymphocytes and IgG^+^CD45R^+^ were determined as B cells. Representative scatter plots and the frequencies and counts of cells in a cardiac allograft (**A**–**F**) and host spleen (**G**–**L**). Data are the mean ± SD of five independent samples. n = 5; *P < 0.05; **P < 0.01

To further explore infiltration by inflammatory cells in grafts, we carried out flow cytometry in the cardiac allograft. The percentages and absolute numbers of CD45^+^ lymphocytes were lower in FGIN1-27-treated and Ro5-4864-treated hearts than those in the hearts of rats in the control group (Fig. 5A–C). The same phenomenon was observed in CD45R^+^IgG^+^ secretion from B cells gated on CD45^+^ cells in the cardiac graft (Fig. 5D–F). Treatment with FGIN1-27 or Ro5-4864 did not reduce the percentages of CD45^+^ lymphocytes or CD45R^+^IgG^+^ B cells in the host spleen but reduced the absolute numbers of them markedly (Fig. 5G–L).

### TSPO ligands reduce the number of infiltrating T cells and macrophages in cardiac grafts

**Fig. 6:**
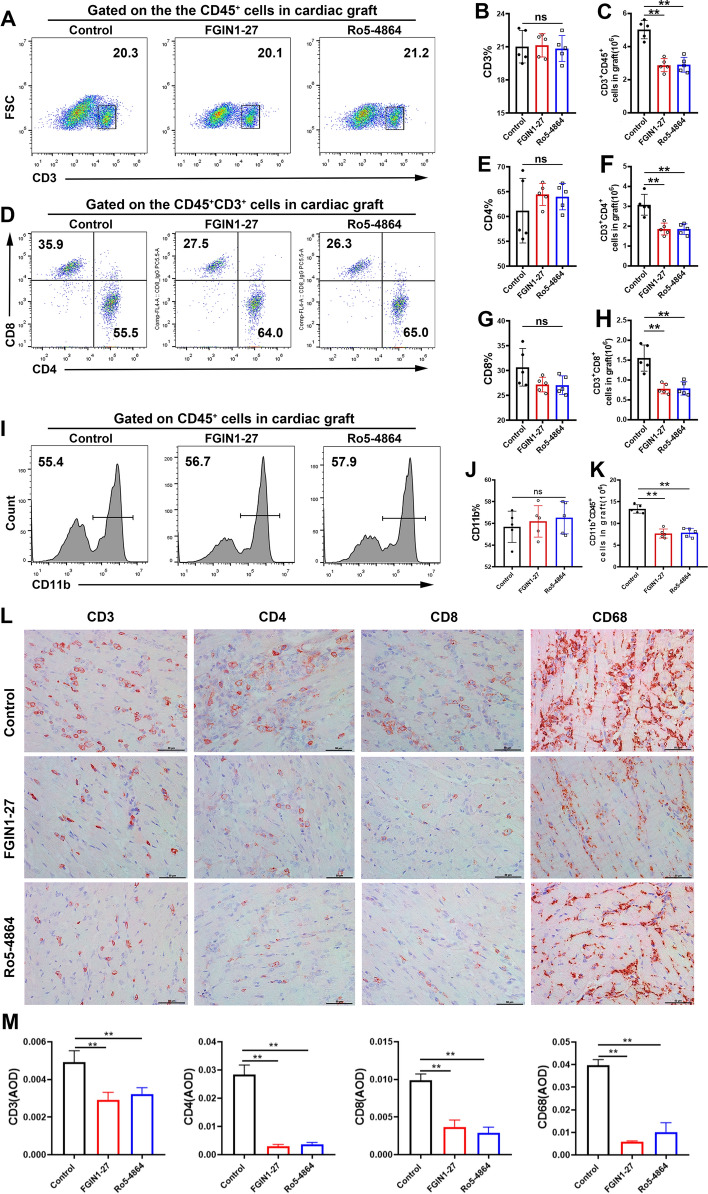
TSPO ligands reduce infiltration by T cells and macrophages in cardiac grafts. **A**–**H** Representative scatter plots and the frequencies and counts of CD3^+^ T cells, CD3^+^CD4^+^ T cells, and CD3^+^CD8^+^ T cells in cardiac allografts. **I**–**K** Representative scatter plots and the frequencies and counts of CD11b^+^ macrophages in cardiac grafts. **L** Immunohistochemical staining of CD3, CD4, CD8, and CD68 in cardiac allografts. **M** AOD of CD3, CD4, CD8, and CD68 staining. Magnification: 400×. n = 5; *P < 0.05; **P < 0.01

Graft-infiltrating T cells and macrophages 4 days after heart transplantation were evaluated by flow cytometry and IHC analyses. Treatment with FGIN1-27 or Ro5-4864 reduced the numbers of CD3^+^ T cells, CD3^+^CD4^+^ T cells, and CD3^+^CD8^+^ T cells in the cardiac graft markedly (Fig. 6A–H). Treatment with FGIN1-27 or Ro5-4864 reduced the number of CD11b^+^ macrophages (Fig. 6I–K). These outcomes were further confirmed by IHC staining for CD3, CD4, CD8, and CD68 (Fig. 6L, M).

### TSPO ligands inhibit mitochondrial respiration and glycolysis in B cells in vitro

**Fig. 7:**
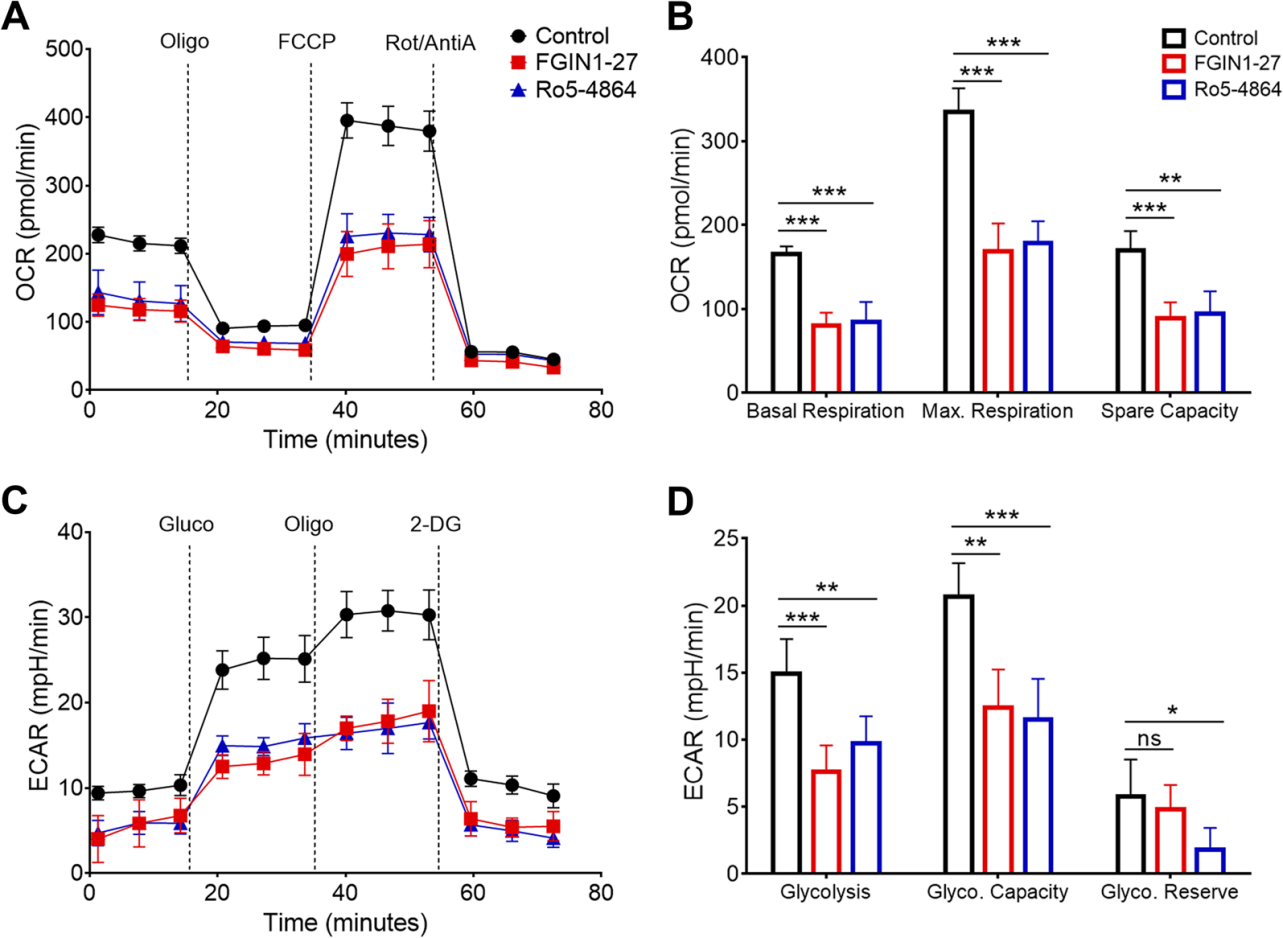
TSPO ligands inhibit mitochondrial respiration and glycolysis in B cells in vitro. **A** The oxygen consumption rate (OCR) of human B cells 48 h after treatment with FGIN1-27 or Ro5-4864. **B** Bar graph of basal and maximal (Max.) respiration of B cells 48 h after treatment with FGIN1-27 or Ro5-4864. **C** The extracellular acidification rate (ECAR) of human B cells 48 h after treatment with FGIN1-27 or Ro5-4864. **D** Bar graph of glycolysis and glycolytic (Glyco.) capacity of B cells 48 h after treatment with FGIN1-27 or Ro5-4864. n = 5; *P < 0.05; **P < 0.01

TSPO is located on the outer mitochondrial membrane and is responsible for respiration and electron transport in mitochondria. We speculated that TSPO ligands may disturb the cellular respiration and metabolism mediated by mitochondria. We assessed the effect of TSPO ligands on the metabolic capacity of B cells using the Seahorse XF assay. The oxidative phosphorylation (OXPHOS) and aerobic glycolysis stress was respectively determined by OCR and ECAR. In the presence of FGIN1-27 or Ro5-4864, OXPHOS was impaired profoundly in activated B cells. Basal respiration, maximal respiration, and spare respiratory capacity were significantly different after treatment (Fig. 7A, B). Aerobic glycolysis stress was also impaired severely in activated B cells in the presence of FGIN1-27 or Ro5-4864 (Fig. 7C, D). These data corroborated our hypothesis that TSPO ligand-mediated mitochondrial respiration was crucial during B cell activation.

### TSPO ligands decrease levels of molecules associated with mitochondrial metabolism

**Fig. 8:**
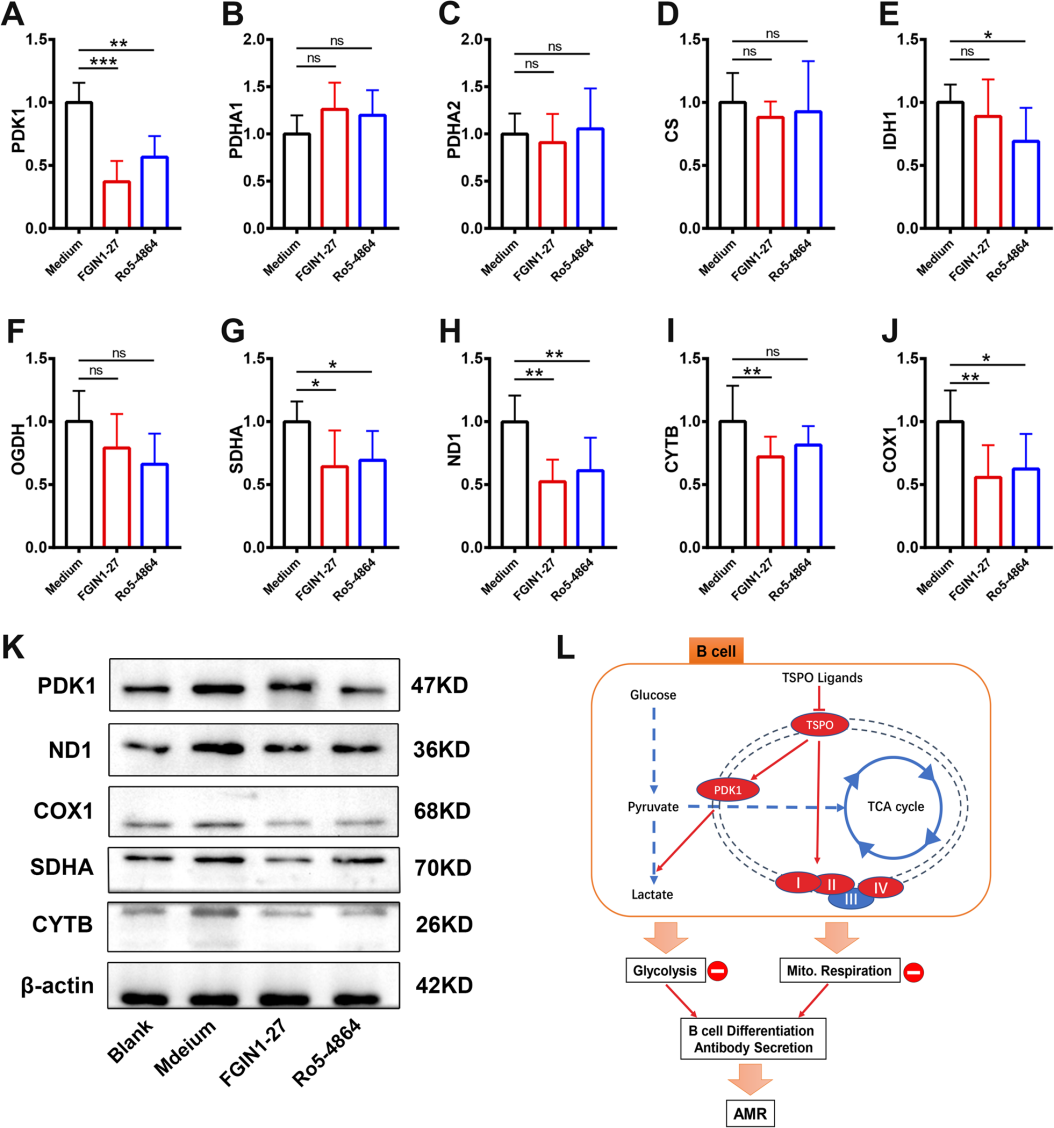
TSPO ligands reduce expression of metabolically relevant molecules in B cells. **A**–**J** mRNA expression of metabolically relevant molecules in mitochondria was determined by real-time RT-qPCR. mRNA expression was normalized to that of β-actin. **K** Protein expression of PDK1, ND1, COX1, SDHA, and CYTB was determined by western blotting. **L** Effect of TSPO ligands on B cells (schematic). n = 5; *P < 0.05; **P < 0.01

To gain insight into the molecular mechanism by which TSPO ligands inhibit mitochondrial respiration and glycolysis in B cells, we searched for the metabolic targets of TSPO ligands. RT-qPCR was used to measure mRNA expression of mitochondrial metabolism-related molecules, including pyruvate dehydrogenase complex (PDH), PDK1, catalytic enzymes in the tricarboxylic acid cycle, citrate synthase, isocitrate dehydrogenase, oxoglutarate dehydrogenase, as well as mitochondrial ND1, COX1, SDHA, and CYTB. Treatment with FGIN1-27 or Ro5-4864 inhibited expression of *PDK1, SDHA, ND1*, and *COX1* in activated B cells (Fig. 8A–J), and western blotting confirmed this scenario at the protein level (Fig. 8K).

Taken together, we confirmed that TSPO ligands interrupted the ETC by inhibiting the transmembrane protein complex proteins SDHA, ND1, and COX1 and obstructing glycolysis by suppressing PDK1 expression.

## Disscussion

AMR is the most serious complication after organ transplantation and is the main cause of immune-mediated graft failure. Specific drug treatment for AMR is lacking. Activated B cells and DSA production are the main reasons for AMR. Expression of TSPO (located in the outer mitochondrial membrane) is believed to be highly upregulated in many inflammatory diseases and immune cells. ​TSPO ligands have also been reported for use in PET imaging of inflammatory disease [19] and as a therapeutic option in the treatment of lymphocytic leukemia [20]. These evidence provide important clues regarding the physiological function of TSPO ligands in AMR.

In this study, we demonstrated that TSPO had high expression in activated B cells and that treatment with TSPO ligands attenuated AMR after allograft transplantation by inhibiting the differentiation and antibody secretion of B cells. Due to the close relationship between mitochondrial respiration and TSPO, we first demonstrated that TSPO ligands suppressed B-cell effector functions by downregulating expression of PDK1 and factors in ETC complexes I, II, and IV to inhibit mitochondrial respiration and glycolysis in B cells (Fig. 7L). These findings suggest the potential value of TSPO ligands in preventing AMR after organ transplantation.

First, we found that, TSPO ligands, FGIN1-27 and Ro5-4864, inhibited the antibody production by suppressing B cells in multiply pathways. Treatment with FGIN1-27 or Ro5-4864 inhibited the proliferation and activation of B cells significantly, disturbed the differentiation of B cells into CD138^+^ plasma cells, and reduced the production of antibodies, in vitro.

DSAs (including IgG and IgM) are produced mainly by MHC-reactive B cells [21, 22]. Pre-existing IgG (but not IgM) and newly generated donor-specific IgG are associated with rejection and indicates a poor prognosis for graft survival [23, 24]. We established a rat skin-graft model and demonstrated the inhibitory effect of FGIN1-27 and Ro5-4864 upon DSA production.

Afterwards, we set up a mix-antibody mediate rat-heart transplantation model to explore the TSPO ligands effect in attenuate DSA-mediated allograft injury. We found that, treatment with FGIN1-27 or Ro5-4864 inhibited the increasing trend in circulating IgG levels significantly. Furthermore, after treatment with FGIN1-27 or Ro5-4864, the duration of survival of cardiac grafts nearly doubled. In addition, the pathologic damage to cardiac grafts was attenuated significantly and C4d deposition (which indicates irreversible damage to a graft) was reduced after treatment with TSPO ligands. These data indicate that TSPO ligands could reduce the tissue damage that improves the prognosis of AMR.

AMR is caused mainly by the activation and antibody secretion of donor-specific B cells [2, 25, 26]. In the heart-transplantation model, the proportion and absolute number of B cells in grafts after treatment with FGIN1-27- or Ro5-4864 were lower than those from the control group. Antibody-secreting plasma cells are the terminally differentiated forms of B cells that carry out effector functions [27–29]. Combined with the evidence of reduced circulating levels of IgG and C4d deposition in grafts, this finding strongly suggested that TSPO ligands inhibited the activation and antibody secretion of B cells significantly in vivo.

Although we used the CSA to reduce the disturbance of T cells and macrophages as much as possible, this model inevitably involved cell-mediated rejection but mainly antibody-mediated rejection. Except for the deposition of complement, we tested for infiltration by inflammatory cells. Secreting B cells (CD45R^+^IgG^+^), but also the numbers of CD3^+^ T cells and CD11b^+^ macrophages, were reduced after treatment with TSPO ligands. In accordance with other studies and our previous results, that the inhibition of TSPO ligands is not specific for one type of lymphocyte, and that the mechanism may be relevant for all types of lymphocytes.

TSPO is a part of mitochondrial membrane transition pore and associated with various mitochondrial functions. One study showed that TSPO knockout impaired mitochondrial oxidative respiration and glycolysis in microglia [30] and TSPO knockouts microglia produce significantly less ATP, suggesting reduced metabolic activity [31]. Mitochondria are the protagonists of cellular energy metabolism [32–34] and target organelles for TSPO ligands. We discovered that TSPO ligands reduced the basal and spare respiratory capacities of B cells significantly. A glycolysis stress test demonstrated that TSPO ligands reduced the glycolysis capacity of B cells significantly. These data suggest that TSPO ligands reduce energy metabolism in B cells by inhibiting mitochondrial respiration and glycolysis.

The energy demands of B cells vary according to the different stages of proliferation and differentiation [35]. If B cells are activated, the levels of mitochondrial respiration and glycolysis increase. Plasma cells have relatively higher metabolic demands because they require high levels of energy and nutrients for antibody secretion [36, 37]. Therefore, the ability of TSPO ligands to reduce the metabolic output in B cells might be the main mechanism underlying their inhibition of B-cell activation, plasma-cell formation, and antibody secretion.

PDH facilitates pyruvate entry into mitochondria [38] and catalyzes the conversion of pyruvate to acetyl coenzyme A [39]. PDH is phosphorylated by PDK1. We found that treatment with TSPO ligands downregulated PDK1 expression significantly after B-cell activation. Therefore, the reduction in glycolysis upon treatment with TSPO ligands was likely due to inhibition of PDK1 expression.

We next discovered that TSPO ligands blocked metabolism in B cells by inhibiting expression of factors in the ETC (ND1, SDHA, COX1). The ETC is composed mainly of electron-transferring protein complexes I, II, and IV [40, 41] located on the inner membrane of mitochondria, and are vital for oxidative phosphorylation [42–45]. The loss or downregulation of expression of these proteins affects mitochondrial oxidative respiration directly and leads to reduced production of adenosine triphosphate [46, 47]. We demonstrated that TSPO ligands reduced mitochondrial respiration in B cells by inhibiting expression of complex molecules in the respiratory chain.

## Conclusions

In summary, we found that TSPO ligands, FGIN1-27 and Ro5-4864, inhibited the B cell differentiation and proliferation in vitro and suppressed the DSA production in vivo. Subsequently, we provide evidence that the significant role of TSPO ligands in prolonging the survival and attenuating injury of cardiac allografts in a mixed model of AMR. We also clarified the internal mechanism underlying the effect of treatment by TSPO ligands in B cells may through blunting expression of the glycolysis-regulating molecule PDK1 and ETC proteins, thereby reducing the B cell glycolytic capacity and mitochondrial respiration capacity. Importantly, our study provides novel drug targets for the clinical treatment of AMR after transplantation.

## Supplementary Information

**Additional file 1: Figure S1**. The pre-sensitized model exhibited a typical AMR presentation. **FigureS2**. B cells expressed TSPO. **Figure S3**. TSPO ligands do not inducedthe B cells apoptosis under the concentration of 100 μM.

## Data Availability

All data are available in the manuscript and Additonal file data.

## Abbreviations

TSPO: 18-kDa translocator protein
AMR: Antibody-mediate rejection
DSAs: Donor-specific antibodies
C4d: Complement 4d
CSA: Cyclosporin A
IgG: Immunoglobulin G
IgM: Immunoglobulin M
MHC: Major histocompatibility complex
PDK1: Pyruvate dehydrogenase kinase 1
ND1: NADH-ubiquinone oxidoreductase chain 1
COX1: Cytochrome C oxidase I
SDHA: Succinate dehydrogenase subunit alpha
CYTB: Cytochrome B
PDHA: Pyruvate dehydrogenase complex
TCA: Tricarboxylic acid
ETC: Electron transport chain
AOD: Average optical density
IHC: Immunohistochemical

## References

[1] Loupy A, Lefaucheur C (2018). Antibody-mediated rejection of solid-organ allografts. N Engl J Med.

[2] Chong AS, Rothstein DM, Safa K, Riella LV (2019). Outstanding questions in transplantation: B cells, alloantibodies, and humoral rejection. Am J Transplant.

[3] Bouquegneau A, Loheac C, Aubert O, Bouatou Y, Viglietti D, Empana JP (2018). Complement-activating donor-specific anti-HLA antibodies and solid organ transplant survival: a systematic review and meta-analysis. PLoS Med.

[4] Kim M, Martin ST, Townsend KR, Gabardi S (2014). Antibody-mediated rejection in kidney transplantation: a review of pathophysiology, diagnosis, and treatment options. Pharmacotherapy.

[5] Jordan SC, Ammerman N, Choi J, Huang E, Peng A, Sethi S (2020). The role of novel therapeutic approaches for prevention of allosensitization and antibody-mediated rejection. Am J Transplant.

[6] Chehade H, Pascual M (2016). The challenge of Acute antibody-mediated rejection in kidney transplantation. Transplantation.

[7] Jordan SC, Toyoda M, Kahwaji J, Vo AA (2011). Clinical aspects of intravenous immunoglobulin use in solid organ transplant recipients. Am J Transplant.

[8] Papadopoulos V, Baraldi M, Guilarte TR, Knudsen TB, Lacapere JJ, Lindemann P (2006). Translocator protein (18 kDa): new nomenclature for the peripheral-type benzodiazepine receptor based on its structure and molecular function. Trends Pharmacol Sci.

[9] Rupprecht R, Papadopoulos V, Rammes G, Baghai TC, Fan J, Akula N (2010). Translocator protein (18 kDa) (TSPO) as a therapeutic target for neurological and psychiatric disorders. Nat Rev Drug Discov.

[10] Zavala F, Taupin V, Descamps-Latscha B (1990). In vivo treatment with benzodiazepines inhibits murine phagocyte oxidative metabolism and production of interleukin 1, tumor necrosis factor and interleukin-6. J Pharmacol Exp Ther.

[11] Daugherty DJ, Selvaraj V, Chechneva OV, Liu XB, Pleasure DE, Deng W (2013). A TSPO ligand is protective in a mouse model of multiple sclerosis. EMBO Mol Med.

[12] Zhang Y, Yu S, Li X, Yang B, Wu C (2017). The ligands of translocator protein inhibit human Th1 responses and the rejection of murine skin allografts. Clin Sci (Lond).

[13] Faas MM, de Vos P (2020). Mitochondrial function in immune cells in health and disease. Biochim Biophys Acta Mol Basis Dis.

[14] Ye L, Jiang Y, Zhang M (2022). Crosstalk between glucose metabolism, lactate production and immune response modulation. Cytokine Growth Factor Rev.

[15] Magri A, Lipari CLR, Risiglione P, Zimbone S, Guarino F, Caccamo A (2023). ERK1/2-dependent TSPO overactivation associates with the loss of mitophagy and mitochondrial respiration in ALS. Cell Death Dis.

[16] Fu Y, Wang D, Wang H, Cai M, Li C, Zhang X (2020). TSPO deficiency induces mitochondrial dysfunction, leading to hypoxia, angiogenesis, and a growth-promoting metabolic shift toward glycolysis in glioblastoma. Neuro Oncol.

[17] Liao T, Li Q, Zhang Y, Yang Z, Huang Z, Han F (2020). Precise treatment of acute antibody-mediated cardiac allograft rejection in rats using C4d-targeted microbubbles loaded with nitric oxide. J Heart Lung Transplant.

[18] Liao T, Liu X, Ren J, Zhang H, Zheng H, Li X (2018). Noninvasive and quantitative measurement of C4d deposition for the diagnosis of antibody-mediated cardiac allograft rejection. EBioMedicine.

[19] Singhal T, Weiner HL, Bakshi R (2017). TSPO-PET imaging to assess cerebral microglial activation in multiple sclerosis. Semin Neurol.

[20] Santidrian AF, Cosialls AM, Coll-Mulet L, Iglesias-Serret D, de Frias M, Gonzalez-Girones DM (2007). The potential anticancer agent PK11195 induces apoptosis irrespective of p53 and ATM status in chronic lymphocytic leukemia cells. Haematologica.

[21] McKenna RM, Takemoto SK, Terasaki PI (2000). Anti-HLA antibodies after solid organ transplantation. Transplantation.

[22] Zhang R (2018). Donor-specific antibodies in kidney transplant recipients. Clin J Am Soc Nephrol.

[23] Abe M, Kawai T, Futatsuyama K, Tanabe K, Fuchinoue S, Teraoka S (1997). Postoperative production of anti-donor antibody and chronic rejection in renal transplantation. Transplantation.

[24] Scornik JC, Salomon DR, Lim PB, Howard RJ, Pfaff WW (1989). Posttransplant antidonor antibodies and graft rejection. Evaluation by two-color flow cytometry. Transplantation.

[25] Karahan GE, Claas FH, Heidt S (2016). B cell immunity in solid organ transplantation. Front Immunol.

[26] Chong AS, Ansari MJ (2018). Heterogeneity of memory B cells. Am J Transplant.

[27] Shinnakasu R, Kurosaki T (2017). Regulation of memory B and plasma cell differentiation. Curr Opin Immunol.

[28] Suan D, Sundling C, Brink R (2017). Plasma cell and memory B cell differentiation from the germinal center. Curr Opin Immunol.

[29] Calame KL (2001). Plasma cells: finding new light at the end of B cell development. Nat Immunol.

[30] Yao R, Pan R, Shang C, Li X, Cheng J, Xu J (2020). Translocator protein 18 kDa (TSPO) Deficiency inhibits microglial activation and impairs mitochondrial function. Front Pharmacol.

[31] Banati RB, Middleton RJ, Chan R, Hatty CR, Kam WW, Quin C (2014). Positron emission tomography and functional characterization of a complete PBR/TSPO knockout. Nat Commun.

[32] Russell OM, Gorman GS, Lightowlers RN, Turnbull DM (2020). Mitochondrial Diseases: hope for the future. Cell.

[33] Chakrabarty RP, Chandel NS (2021). Mitochondria as Signaling Organelles Control mammalian stem cell fate. Cell Stem Cell.

[34] Carrico C, Meyer JG, He W, Gibson BW, Verdin E (2018). The mitochondrial acylome emerges: proteomics, regulation by Sirtuins, and metabolic and Disease Implications. Cell Metab.

[35] Boothby M, Rickert RC (2017). Metabolic regulation of the Immune Humoral Response. Immunity.

[36] Aronov M, Tirosh B (2016). Metabolic control of plasma cell differentiation- what we know and what we don’t know. J Clin Immunol.

[37] Guo M, Price MJ, Patterson DG, Barwick BG, Haines RR, Kania AK (2018). EZH2 represses the B Cell Transcriptional Program and regulates antibody-secreting cell metabolism and antibody production. J Immunol.

[38] Patel MS, Roche TE (1990). Molecular biology and biochemistry of pyruvate dehydrogenase complexes. FASEB J.

[39] Patel MS, Nemeria NS, Furey W, Jordan F (2014). The pyruvate dehydrogenase complexes: structure-based function and regulation. J Biol Chem.

[40] Lobo-Jarne T, Ugalde C (2018). Respiratory chain supercomplexes: structures, function and biogenesis. Semin Cell Dev Biol.

[41] Rich PR, Marechal A (2010). The mitochondrial respiratory chain. Essays Biochem.

[42] Cecchini G (2003). Function and structure of complex II of the respiratory chain. Annu Rev Biochem.

[43] Hosler JP, Ferguson-Miller S, Mills DA (2006). Energy transduction: proton transfer through the respiratory complexes. Annu Rev Biochem.

[44] Sazanov LA (2015). A giant molecular proton pump: structure and mechanism of respiratory complex I. Nat Rev Mol Cell Biol.

[45] Hatefi Y (1985). The mitochondrial electron transport and oxidative phosphorylation system. Annu Rev Biochem.

[46] Gehrke S, Wu Z, Klinkenberg M, Sun Y, Auburger G, Guo S (2015). PINK1 and parkin control localized translation of respiratory chain component mRNAs on mitochondria outer membrane. Cell Metab.

[47] Guerrero-Castillo S, Baertling F, Kownatzki D, Wessels HJ, Arnold S, Brandt U (2017). The Assembly pathway of mitochondrial respiratory chain complex I. Cell Metab.

