# Supplementary material for: Preventative effect of TSPO ligands on mixed antibody-mediated rejection through a Mitochondria-mediated metabolic disorder
Source: J Transl Med. 2023 May 2;21:295. doi: 10.1186/s12967-023-04134-2 (PMC10152746; doi:10.1186/s12967-023-04134-2)
Supplement: Supplementary file 1 — Additional file 1: Figure S1. The pre-sensitized model exhibited a typical AMR presentation. FigureS2. B cells expressed TSPO. Figure S3. TSPO ligands do not inducedthe B cells apoptosis under the concentration of 100 μM. [file 12967_2023_4134_MOESM1_ESM.docx]

**Additional file material:**


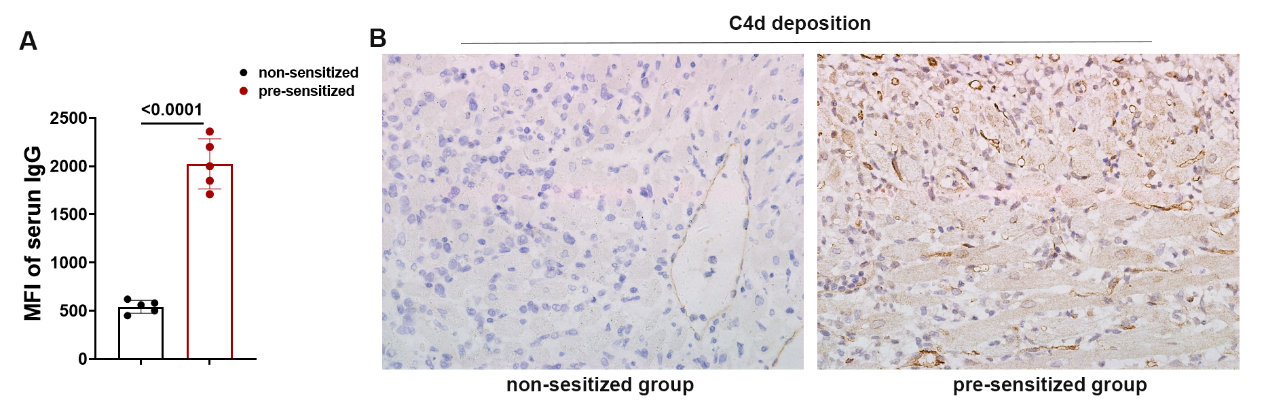


**Additional file 1: Figure S1. The pre-sensitized model exhibited a typical AMR presentation.** Rats were randomly divided into two groups: non-sensitized group only performed the cardiac transplantation; pre-sensitized group received the allo-hearts 14 days after skin transplantation. The recipients were all executed at day 4 after heart transplantation (A)The circulating donor specific-IgG were determined by FACs. (B) C4d depositions in graft were determined by IHC. (n=5)

**
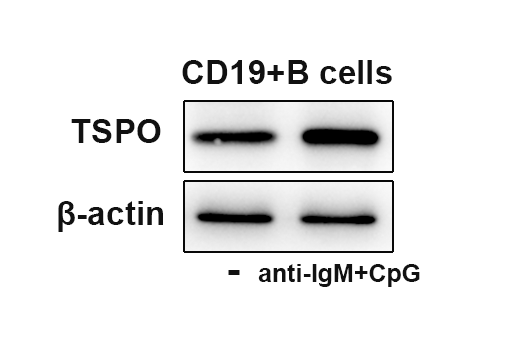
**

**Additional file 1: Figure S2. B cells expressed TSPO.** Purified CD19+B cells were purified from mouse splenocytes and activated with anti-IgM plus CpG. The expression of TSPO in both rest and activated B cell was tested by WB.


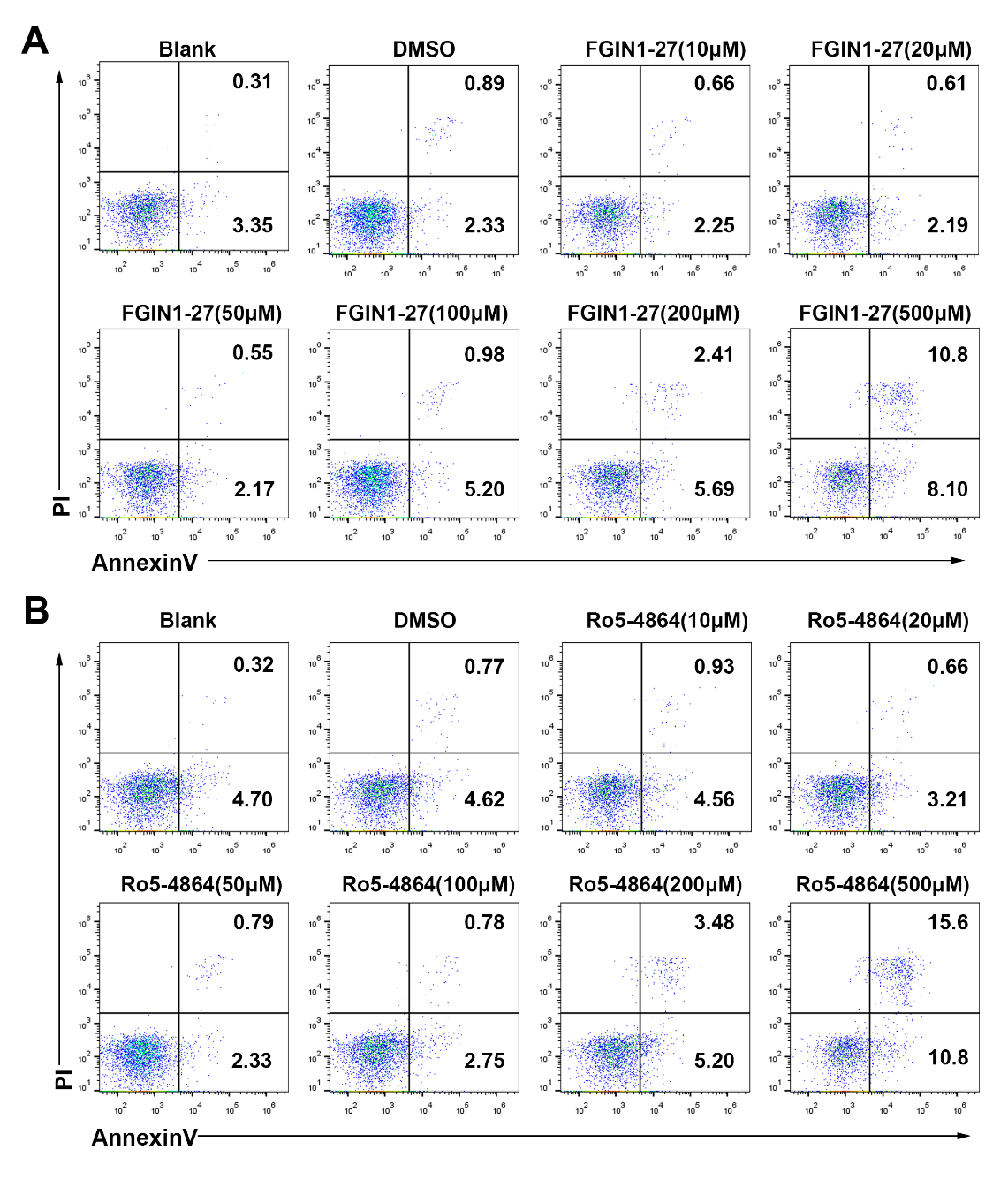
 **Additional file 1: Figure S3. TSPO ligands do not induced the B cells apoptosis under the concentration of 100μM.** (A and B) Purified CD19+ B cells were cultured with or without different concentrations (10, 20, 50, 100, 200, 500 μM) of TSPO ligands, FGIN1-27 or Ro5-4864. Flow cytometry analysis of the Annexin V/PI staining was used for the apoptosis of B cells.
